# Holiday trip to Norway — a stable isotope project on hair strands of individuals of a travel group from Bavaria

**DOI:** 10.1007/s00414-022-02839-5

**Published:** 2022-06-04

**Authors:** Christine Lehn, Annika Hameder, Matthias Graw

**Affiliations:** Institute of Legal Medicine, Ludwig-Maximilians-University of Munich, 80336 Munich, Germany

**Keywords:** Stable isotopes, Bio-elements (C, N, S, H), Hair segments, Diet, Travelling

## Abstract

Hair strands were taken from individuals of a travel group from Bavaria that stayed on the Lofoten Islands/Norway for 3 weeks. By means of serial stable isotope analyses of carbon, nitrogen, sulphur and hydrogen along the hair strands, food-specific changes during travel could be detected. The higher consumption of marine fish led to significant changes of the stable isotope values of nitrogen, sulphur and hydrogen. The highest differences for the values were found in the most proximal part of hair strands which were taken shortly after the trip. The basic values for the isotope distribution of the elements in the hair also indicate specific diets of some individuals that could be confirmed upon request.

## Introduction

Multi-elemental isotope investigations are used to determine provenance and living circumstances of unknown corpses in forensics [1–5]. Stable isotope analyses of the elements carbon (^13^C/^12^C), nitrogen (^15^N/^14^N), sulphur (^34^S/^32^S) and hydrogen (^2^H/^1^H) in the human body tissues provide information about the composition of food and the geographical whereabouts from childhood to death. Information about the last weeks and months of a person’s life is contained in the continuously growing hair. Changes in the nutritional basis within this period can be detected by stable isotope analyses at individual sections along a hair strand.

The isotopic information from solid and liquid food is incorporated into the hair keratin within 1–2 days after food intake [6, 7]. As the mean monthly grow rate of human scalp hairs are ~ 1.1 (± 0.3) cm, hair segments with a length of 2–3 mm span the information over a lifetime of about 1 week. As a result, even short periods associated with a change in diet should be recognizable by a shift in the stable isotope values in the hair segments. A controlled dietary change for 28 days, including a change from C3 to C4 plant enriched diets and a simultaneous replacement of terrestrial animal product by marine products resulted in a significant increase of *δ*^13^C_hair_ and *δ*^15^N_hair_ values of all individuals, although no subject reached a new steady state for either carbon or nitrogen [8].

A vacation trip is often associated with a change of location to another climatic region and with a change in individual eating habits or the composition of the food at the vacation destination. During a trip to the sea coast with an increasing consumption of sea fish, an increase of the sulphur, nitrogen and hydrogen isotope values in the body tissues of the persons can be expected.

In this study the effects of a 3-week stay on the Lofoten/Norway on the stable isotope values of carbon, nitrogen, sulphur and hydrogen in the hair of a travel group from the Munich area were investigated, supplemented by personal information of the travellers.

## Material and methods

In the case of 7 travellers from Bavaria, individual strands of hair were cut off as close to the scalp as possible 3–14 weeks after the trip (for organisational reasons at different times). From one test person, 3 strands of hair were taken at different intervals (20, 64 and 97 days after the trip). After cutting off a hair strand, the most proximal hair section is remaining on the head. Even if an attempt was made to cut off the hair directly at the scalp, we must assume that from 0.5 to 1 cm of the hair root section remain on the test persons’ head [9].

### Sample preparation

**Fig. 1:**
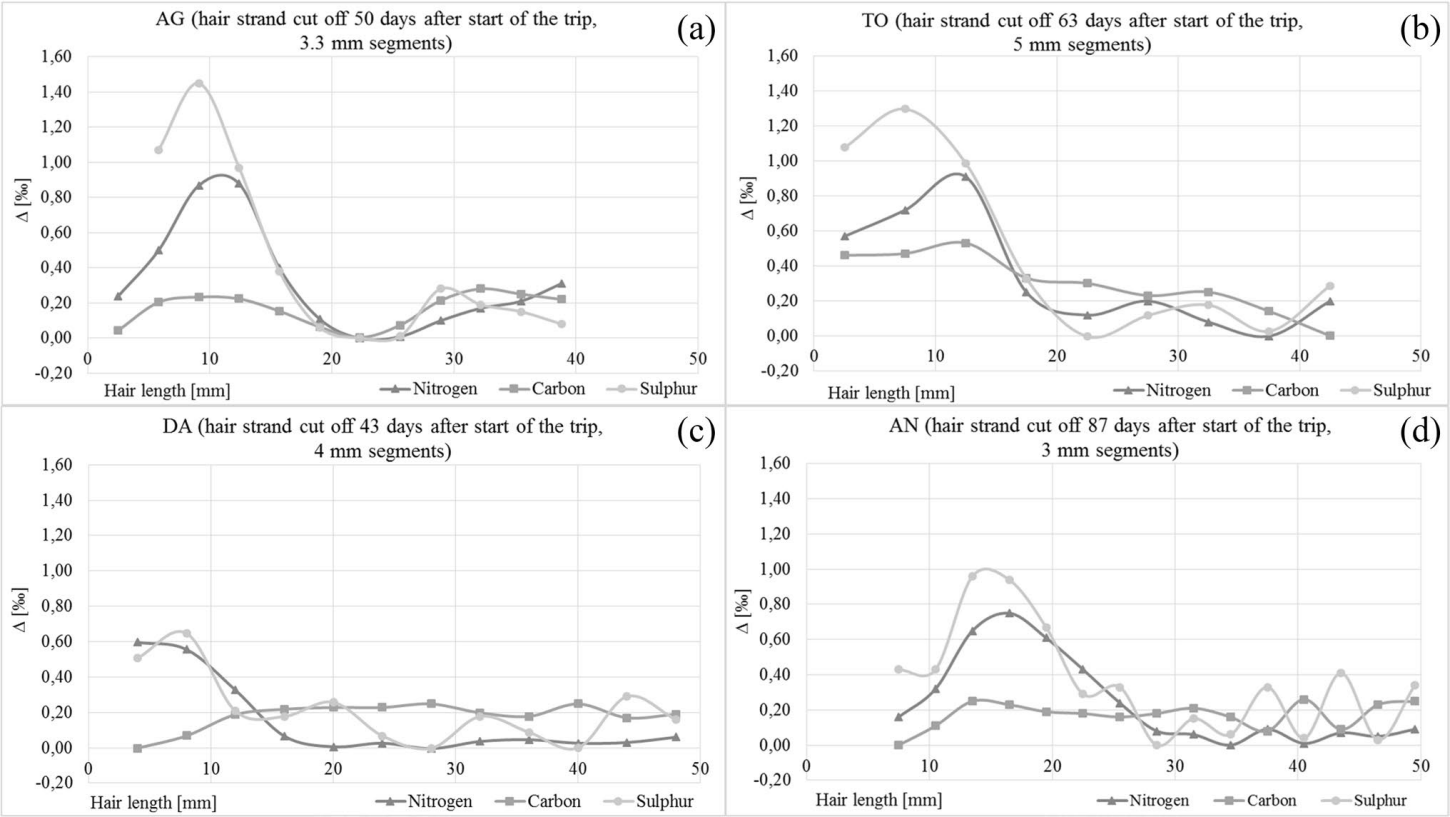
Difference between the highest and the lowest isotope values for nitrogen, carbon and sulphur in the hair segments of AG (**a**), TO (**b**), DA (**c**) and AN (**d**)

**Fig. 2:**
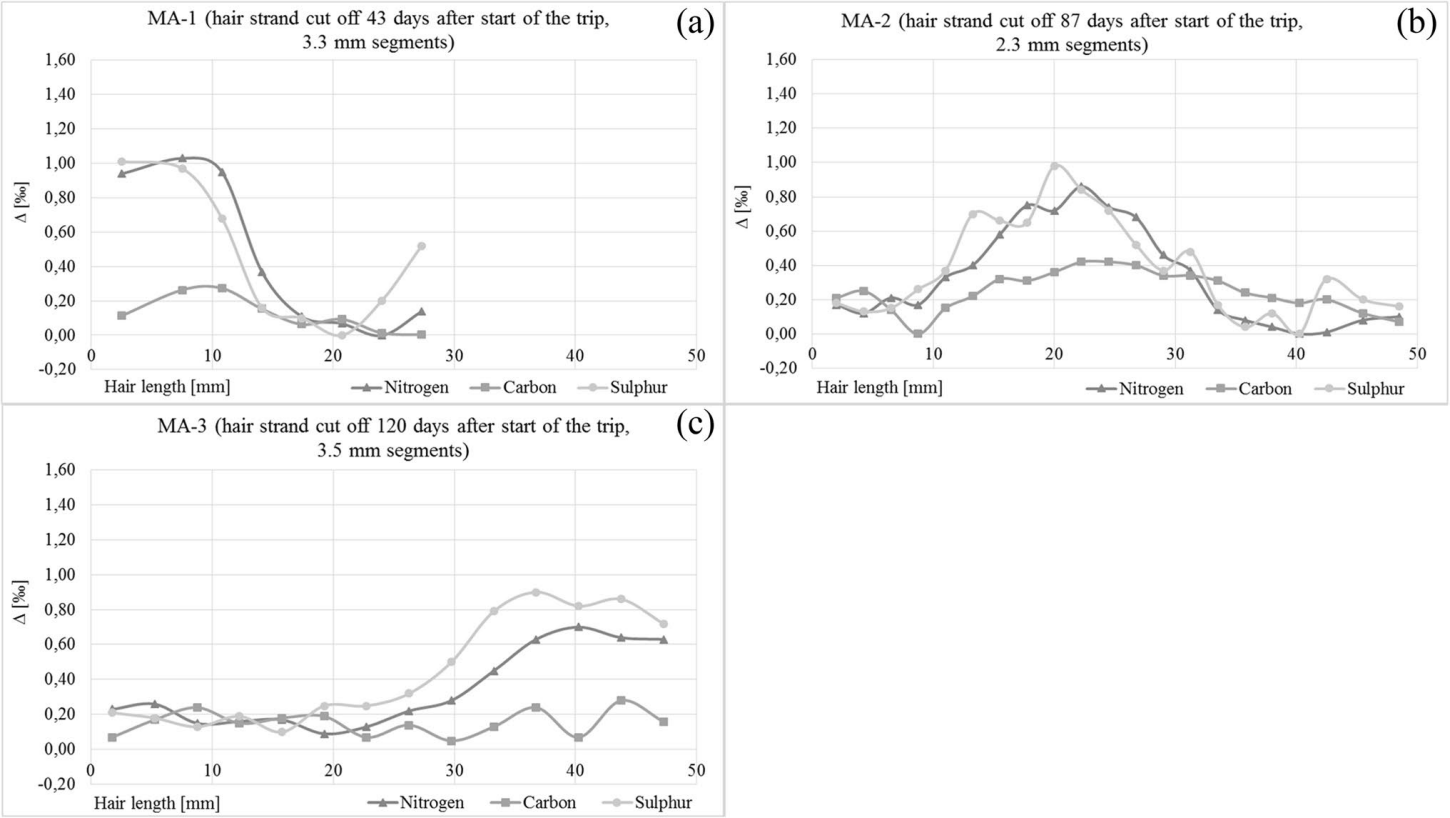
Difference between the highest and the lowest isotope values for nitrogen, carbon and sulphur in the hair segments of MA-1 (**a**), MA-2 (**b**), and MA-3 (**c**)

The hair strands were segmented into short sections of 2.3 to 5 mm in length (see Figs. 1 and 2), depending on the thickness of the hair strand and thus on the sufficient amount of material to analyse. The hair segments then were degreased by immersion in methanol/chloroform (2:1) for about 1 h. After drying, the samples were rinsed several times in distilled water and subsequently dried at room temperature. The segments were cut by scissors in pieces < 1 mm.

### Isotope analysis

For simultaneous analyses of C-N-S-isotopes, 1.8–2.0 mg of the hair samples was weighed into tin capsules. For hydrogen isotope analyses, 150 µg was weighed into tin capsules. The consecutive hair segments from the same subject were analysed in one series of measurements. Stable isotope analyses on the hair samples were performed at Isolab GmbH, Schweitenkirchen, Germany.

Stable isotope abundances of the “bio-elements” (H, C, N, O, S) are typically expressed in delta notation (*δ*) as a relative difference of isotope ratios [10]. The ratio (*R*) of the heavier-to-lighter isotope (e.g. ^13^C/^12^C) of a sample is compared to that of an internationally agreed standard: *δ* = (*R*_sample_/*R*_standard_) − 1, where the value usually is given in permil (‰). The zero-point ratios are defined by international standards, for *δ*^13^C values by Vienna Pee Dee Belemnite (VPDB), for *δ*^15^N values by AIR, for *δ*^34^S values by Vienna Canyon Diablo Troilite (VCDT), and for *δ*^2^H values by Vienna Standard Mean Ocean Water (VSMOW).

Bulk stable isotope ratios of the bio-elements (*δ*^13^C, *δ*^15^N and *δ*^34^S) in the hair samples were analysed according to Sieper et al. [11]. Measurement was carried out using an elemental analyzer–isotope-ratio mass spectrometer (EA–IRMS); for C–N–S simultaneous analysis, an Elementar Vario Cube EL (Elementar Analysensysteme GmbH, Hanau, Germany) connected with an Isoprime mass spectrometer (Isoprime Ltd. Cheadle Hulme, UK) was used; for H isotope analysis, a Thermo high temperature conversion unit connected with a Thermo XP plus IRMS (Thermo Fisher, Bremen, Germany) was used.

Internal standards used for calibration were casein and two different horse tail hair samples (horse tail hair from Bavaria (*δ*^13^C: − 25.61 ‰ (± 0.10), *δ*^15^N: 5.04 ‰ (± 0.09), *δ*^34^S: 6.88‰ (± 0.49), 45 series of measurement) and from Paraguay (*δ*^13^C: − 17.83 ‰ (± 0.17), *δ*^15^N: 6.70‰ (± 0.12), *δ*^34^S: 10.27‰ (± 0.55), 45 series of measurement). For *δ*^15^N and *δ*^34^S, scale calibrations with inorganic reference materials were performed (IAEA-NO-3 and USGS25 for *δ*^15^N; IAEA-S-1, IAEA-SO-5 and IAEA-SO-6 for *δ*^34^S). Scale calibration for *δ*^13^C was performed with organic materials (NBS 22 (oil) and IRMM-BCR 657 (glucose)) [12].

For stable isotope analysis of *δ*^2^H, hair samples were analysed according to the comparative equilibration method [13]. Hair samples and laboratory reference materials were stored under identical conditions for at least 3 days before analysis to enable hydrogen exchange with hydrogen from ambient air moisture. After equilibration, the samples were dried under a vacuum for at least 24 h to remove all adhering humidity. The *δ*^2^H standards also comprised casein and two samples of horse tail hair from Paraguay and Bavaria with *δ*^2^H values of − 55.8‰ (± 3.6) and − 83.8‰ (± 3.1) (50 series of measurement), respectively, which had been calibrated against official human hair reference samples (USGS42 and USGS43) [14, 15]. Samples and the standards were loaded into the helium flushed auto sampler. Hydrogen gas produced by high temperature conversion at 1400 °C was analysed isotopically. The standards were used to normalise measured sample values with the normalization being from 5 to 10‰. International reference material for the calibration of the hydrogen reference gas was NBS 22 with the assigned value of − 120‰ vs. VSMOW.

Analytical uncertainties were ± 0.1‰ for *δ*^13^C_VPDB_, ± 0.2‰ for *δ*^15^N_AIR_, ± 0.3‰ for *δ*^34^S_VCDT_, and ± 3‰ for *δ*^2^H_VSMOW_ values.

## Results

The scalp hair strands were taken from 19- to 61-year old females (4) and males (3) between 43 and 120 days after the trip to Norway started (Table 1). The isotope values in the hair segments grown before the journey are considered to be the home values, reflecting the individual eating habits at the persons’ permanent place of residence. Stable isotope analyses indicated significant changes in the isotope values along the hair strands (Figs. 1 and 2). The changes are temporally related to the trip to the Lofoten Islands.

**Table 1:** Details on the test persons, and stable isotope values of nitrogen (*δ*^15^N), carbon (*δ*^13^C), sulphur (*δ*^34^S) and hydrogen (*δ*.^2^H) in the hair sections. Average value of the last 2 months before the trip (Home); maximum value during the trip to Norway (Lof); difference of value between Home and Lof (∆). Date of travel: from 20 July to 15 August (23 days). Mean value in reference hair samples from Germany (GER, *N* = 161) or Norway (NOR, *N* = 8) [2]

Subject	MA-1	MA-2	MA-3	DA	AG	TO	CL	AN	AL	MV ± SD*		MV ± SD [2]
Gender	m			f	f	m	f	f	m			
Age	59			19	60	30	29	31	33			
Hair sampling (days after start of the trip)	43	87	120	43	50	63	63	87	88			
Hair length grown between start of the trip and sample collection (mm)**	20	39	56	20	23	29	29	41	41			
*δ*^15^N Home [‰]	9.29	9.30	9.22	8.87	9.63	9.42	8.33	9.28	8.71	9.08 ± 0.42	GER	8.47 ± 0.57
*δ*^15^N Lof [‰]	10.19	10.05	9.90	9.42	10.28	10.21	8.85	9.98	9.27	9.74 ± 0 0.52	NOR	8.98 ± 0.43
∆ *δ*^15^N [‰]	0.90	0.75	0.68	0.54	0.65	0.79	0.52	0.70	0.56	0.67		0.51
*δ*^13^C Home [‰]	− 20.27	− 20.24	− 20.26	− 19.41	− 19.98	− 19.83	− 21.18	− 20.04	− 20.37	− 20.16 ± 0.51	GER	− 20.82 ± 0.40
*δ*^13^C Lof [‰]	− 20.04	− 20.00	− 20.07	− 19.61	− 20.00	− 19.46	− 21.17	− 19.99	− 20.42	− 20.10 ± 0.52	NOR	− 21.10 ± 0.29
∆ *δ*^13^C [‰]	0.24	0.24	0.19	− 0.20	− 0.02	0.38	0.01	0.05	− 0.05	0.06		− 0.28
*δ*^34^S Home [‰]	8.30	8.26	8.22	7.77	8.05	7.97	7.46	8.11	7.73	7.91 ± 0.26	GER	6.73 ± 0.88
*δ*^34^S Lof [‰]	8.97	8.96	8.99	8.17	9.36	9.12	8.14	8.86	8.86	8.78 ± 0.43	NOR	8.79 ± 1.09
∆ *δ*^34^S [‰]	0.67	0.70	0.77	0.40	1.31	1.15	0.68	0.75	1.14	0.87		2.06
*δ*^2^H Home [‰]	− 65			− 78	− 66	− 71	− 78			− 71.6 ± 5.6	GER	− 74.1 ± 8.9
*δ*^2^H Lof [‰]	− 58			− 75	− 58	− 61	− 70			− 64.4 ± 6.9	NOR	− 71.5 ± 5.8
∆ *δ*^2^H [‰]	7			3	8	10	8			7.2		2.6

^*^Mean value excluding MA-2 and MA-3; **assuming a hair growth rate of 1.4 cm per 30 days [6]

**Fig. 3:**
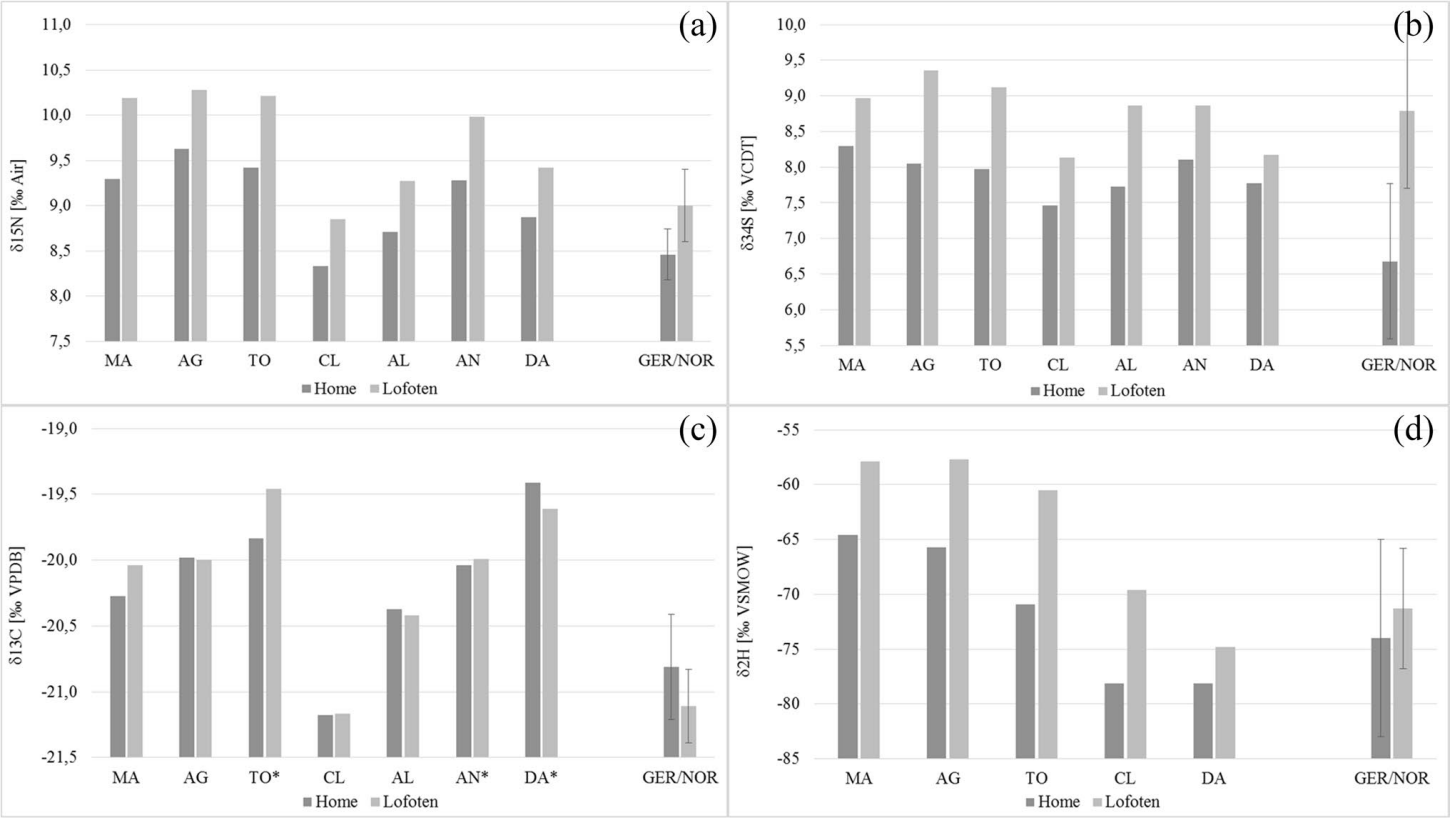
Stable isotope values of nitrogen (**a**), sulphur (**b**), carbon (**c**) and hydrogen (**d**) in the hair segments of the individuals before the trip (Home), at the end of the trip (Lofoten), and the mean values (± SD) in reference hair samples from Germany and from Norway [2]. The individuals with gluten intolerance are marked (asterisk) in the carbon isotope values. Since they partially replaced wheat products (C3 plant) with food based on corn (C4 plant), the carbon isotope values in their hair strands are increased

The home values for nitrogen range from 8.3 to 9.6‰. During the stay in the Lofoten Islands, the *δ*^15^N values increased significantly by up to 0.9‰ (Fig. 3a).

**Fig. 4:**
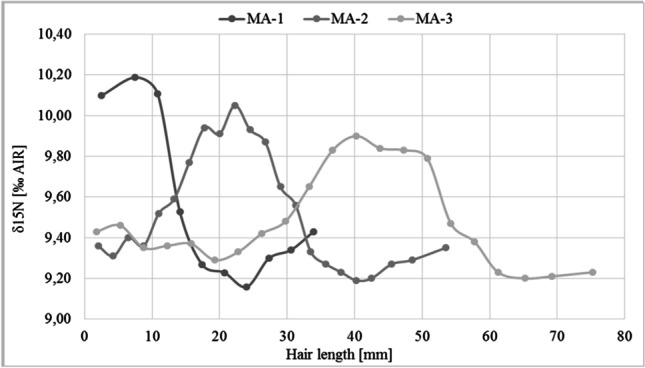
Nitrogen isotope values along the three hair strands taken at different dates after start of the trip to the Lofoten. With increasing distance to the hair root, the peaks become more blurred due to different growth rates of the individual hairs

The home values for sulphur are between 7.5 and 8.3‰, and the stay on the Lofoten Islands led to a significant increase in the *δ*^34^S values in the hair segments of up to 1.3‰ (Fig. 3b).

The basic values for carbon in the hairs segments of the test persons are between − 21.2 and − 19.4‰. During the stay on the Lofoten, the *δ*^13^C values changed only insignificantly for most of the test persons, except for MA and TO, whose value increased, and DA, whose values decreased slightly (Fig. 3c). Reference hair samples from Norway have slightly lower *δ*^13^C values (− 21.1 ± 0.3‰) than those from Germany (− 20.8 ± 0.4‰).

The home values for hydrogen are in the range of − 78 and − 65‰, they correspond to the *δ*^2^H values in reference samples from Germany (− 74 ± 9‰). The stay in the Lofoten led to a significant increase of the *δ*^2^H values by 3 to 10‰ (Fig. 3d).

The 3-week stay on the Lofoten Islands resulted in isotope peaks appearing along the hair strands. Depending on the date of haircut, the peaks were located at different places on the hair strands. The later the hair strand was cut, the more distal the isotopic peak occurred (Fig. 4). The isotopic changes are most pronounced at the root end of the hair strand taken the shortest period after return from the journey (MA-1). In the hair taken at later times, the hair sections influenced by the stay on the Lofoten Islands are located further distal (MA-2, MA-3), and the shape of the peaks become increasingly wider and flatter.

## Discussion

As a result of the dietary changes in the Lofoten Islands, food-specific isotopic shifts in the body tissues of the test persons could be detected by serial analyses on their hair strands. The stay on the Lofoten Islands led to significant increases in the nitrogen and sulphur, as well as in hydrogen isotope ratios. The greatest extent of isotopic changes along the hair strand coincides with the end of the stay on the Lofoten. These changes correspond well with the isotopic differences observed in reference hair samples from Germany and from Norway (Table 1). Reference hair samples from Norway are enriched in ^15^N by 0.5‰, in ^34^S by 2.1‰ and in ^2^H by 3‰ compared to those from Germany.

The pattern of isotopic changes in the hair strands is consistent with an increased consumption of marine animals on the Lofoten. Marine animals are at the highest level of the food chain, as a result their body tissues have very high nitrogen and hydrogen isotope levels [16, 17]. Protein from sea fish has ~ 5.5‰ higher *δ*^13^C values, ~ 7.0‰ higher *δ*^15^N values than protein from terrestrial animals, such as pig or beef [8]. The *δ*^2^H values for carnivores/piscivores differ by about 90‰ from herbivores/omnivores in both terrestrial and aquatic systems [16]. In addition, marine products have the highest *δ*^34^S values within the food spectrum (~ 20‰) [18, 19]. Consequently, body tissues of individuals who consume a lot of marine animals usually have elevated *δ*^15^N, *δ*^34^S, *δ*^13^C and *δ*^2^H values compared to a herbivorous diet [20–22].

If people exchange dietary proteins from terrestrial animals by such from sea fish, corresponding isotopic changes in the hair keratin of the consumers can be expected. Although the isotopic information from food and drinks are incorporated into hair keratin within 1–2 days [7], the final isotopic differences as found between the different food groups are not expected to be reached after 23 days [8]. Furthermore, a single hair segment spans a growth period of ~ 6 to ~ 13 days and hence contains the mean but not the absolute level of isotopic changes in the hair keratin. Although their values are unlikely to have reached steady state level, we can assume that the test persons with the highest nitrogen and sulphur isotope peaks in their hair strands (MA, AG, TO, AN) have received their dietary protein on the Lofoten Islands predominantly from marine animals. It is striking that these very people also have the highest home values for nitrogen and sulphur, which are significantly higher than the mean values for reference hair samples from Germany. This suggests that these individuals have a special preference for marine products in their everyday diets.

Enquiries about the composition of the diet showed that sea angling was a popular leisure activity during the stay on the Lofoten. The success of these efforts was prepared and eaten together almost every day. Sea fish such as stonefish and cod were the main component of the animal food protein in Lofoten. Not only on the Lofoten, but also at home MA and AG eat sea fish 1–2 times a week, which is very unusual at least for South German people.

Noticeable are the different carbon isotope values in the hairs of the individual persons. Most of them have higher home values than the “typical” German value range (− 20.8 ± 0.4‰). The *δ*^13^C values in the hair samples of TO and DA are even at the upper limit of the comparative values found in Germany. This means an above-average amount of maize or cane sugar in their everyday diet, which are usually not common in food products in Central Europe. Maize and sugar cane belong to the group of C4 plants with a relatively high ^13^C abundance, leading to about 14‰ higher *δ*^13^C values compared to C3 plants, the latter including wheat, rye, potatoes and rice, which represent the staple food in most of the European countries. *δ*^13^C values higher than − 20.0 ‰, as found in the hair strands of TO and DA, are rather unusual for people from Germany. Overall, such values are untypical for European and West Asian countries, but are very common in the hair samples from regions where C4 plants are more common in staple food, e.g. North and South America as well as Africa [23, 24]. Thus, it is very likely that this results from a habitual above-average consumption of food products based on C4 plants. There are different causes that may explain the high carbon isotope levels in the hair of TO and DA. On the one hand, the high *δ*^13^C home values in the hair of TO and DA could be the consequence of a longer stay in countries where maize products are part of the basic diet; or they are due to health necessities of the both persons. When asked, TO, AN and DA have indicated that they have an intolerance to gluten. The diagnosis of coeliac disease requires that gluten-containing cereals such as wheat, rye, barley or spelt be largely avoided for health reasons. The carbohydrate requirement can be replaced by gluten-free plant species such as corn, rice, millet, buckwheat or potatoes. TO and DA replaced gluten-containing food products more often with food products based on maize (e.g. pasta), leading to the comparatively high carbon isotope levels in their hair. AN, however, replaces gluten-containing cereals more often with potatoes and rice. As these belong to the C3 plants, this replacement has no impact on the *δ*^13^C values usually characterised by C3 plant nutrition in Germany.

DA not only has the highest *δ*^13^C values, but also the lowest *δ*^34^S and *δ*^2^H values. The overall isotopic picture is the most likely to suggest that the hair of DA is influenced by a longer stay abroad. It turned out that 2 years before the trip to Norway, DA had lived with a family in Vancouver, Canada for 12 months. Reference hair samples from Canada have higher *δ*^13^C (average − 18.1 ± 0.5‰, *N* = 15) and lower *δ*^34^S values (average 4.5 ± 1.3‰, *N* = 15) than those from Germany or Norway [23, 25]. It is known of hair that it incorporates the metabolites from currently consumed food and those from the body’s own metabolism. But this only applies to growing hair (about 80–85% of the single hairs in a hair strand). About 15–20% of hair is in the resting phase, which can last up to 6 months, before the hair falls out [26]. If a person has spent a lot of time abroad, the isotope information resulting from this may be visible in the hair strand, even after the journey [6, 27]. In this case, however, the proximal part of the hair strand should no longer be influenced by the longer past stay in Canada. On the other hand, it must be considered that isotopic information from the previous diet can enter the hair keratin via conversion or metabolic processes of body tissues. It is possible that changes in body weight, combined with a reduction in muscle mass and body fat, have led to the observed shifts in the isotope values of nitrogen and carbon in the hair [5, 28, 29].

Assuming a maximal hair growth rate of 1.4 cm/ 30 days [7], the beginning point of isotopic changes correlate well with the travel dates to Norway, and the 3-week stay on the Lofoten Islands resulted in isotope peaks appearing along the hair strands. On MA’s three hair strands, which were taken at different times after the trip, the peaks are at different distances from the hair root. In addition, the peak height became increasingly lower and the peak width got larger as the distance to the scalp increased. This is in accordance with the observation that due to different growth rates of the individual hairs, travel-related isotope signals become increasingly blurred with greater distance from the hair root [6, 27].

## Conclusion

By means of serial stable isotope analyses of hair strands of volunteers of a travel group from Bavaria, the food-specific changes could be detected during a 3-week stay on the Lofoten/Norway. The higher consumption of marine fish led to significant isotope peaks of nitrogen and sulphur along the hair strands. Additionally, the hydrogen isotope values increased, whereas the carbon isotope values remained almost at a constant level. The highest differences for the values were found in the most proximal part of hair strands which were taken shortly after the trip. In the hair strands taken later, the isotope peaks are further distal and become more and more blurred with increasing distance from the hair root. The basic values for the isotope distribution of the elements in the hair also indicate the specific nutritional habits of the individual test persons, for example the individual consumption of animal protein or the avoidance of gluten-containing foods. The higher carbon isotope levels in the hair can be explained by a gluten-free diet and the consumption of corn-based food products.

The results of the study show the variability of stable isotope data within a group of persons living in the same region. The personal information related to the data may be support data interpretation in forensically relevant cases.
